# Platelet Activating Factor Receptor Exaggerates Microglia-Mediated Microenvironment by IL10-STAT3 Signaling: A Novel Potential Biomarker and Target for Diagnosis and Treatment of Alzheimer’s Disease

**DOI:** 10.3389/fnagi.2022.856628

**Published:** 2022-04-28

**Authors:** Junxiu Liu, Linchi Jiao, Xin Zhong, Weifan Yao, Ke Du, Senxu Lu, Yuqiang Wu, Tianxin Ma, Junhui Tong, Mingyue Xu, Wenjuan Jiang, Yubao Wang, Miao He, Wei Xin, Mingyan Liu

**Affiliations:** ^1^Department of Pharmacology, School of Pharmacy, China Medical University, Shenyang, China; ^2^Liaoning Key Laboratory of Molecular Targeted Anti-Tumor Drug Development and Evaluation, China Medical University, Shenyang, China; ^3^Liaoning Cancer Immune Peptide Drug Engineering Technology Research Center, China Medical University, Shenyang, China; ^4^Key Laboratory of Precision Diagnosis and Treatment of Gastrointestinal Tumors, Ministry of Education, China Medical University, Shenyang, China; ^5^Liaoning Medical Diagnosis and Treatment Center, Shenyang, China; ^6^The First Affiliated Hospital of China Medical University, Shenyang, China

**Keywords:** Alzheimer’s Disease, biomarkers, microglia, microenvironment, PTAFR

## Abstract

**Background:**

Early diagnosis and effective intervention are the keys to delaying the progression of Alzheimer’s Disease (AD). Therefore, we aimed to identify new biomarkers for the early diagnosis of AD through bioinformatic analysis and elucidate the possible underlying mechanisms.

**Methods and Results:**

GSE1297, GSE63063, and GSE110226 datasets from the GEO database were used to screen the highly differentially expressed genes. We identified a potential biomarker, Platelet activating factor receptor (PTAFR), significantly upregulated in the brain tissue, peripheral blood, and cerebrospinal fluid of AD patients. Furthermore, PTAFR levels in the plasma and brain tissues of APP/PS1 mice were significantly elevated. Simultaneously, PTAFR could mediate the inflammatory responses to exaggerate the microenvironment, particularly mediated by the microglia through the IL10-STAT3 pathway. In addition, PTAFR was a putative target of anti-AD compounds, including EGCG, donepezil, curcumin, memantine, and Huperzine A.

**Conclusion:**

PTAFR was a potential biomarker for early AD diagnosis and treatment which correlated with the microglia-mediated microenvironment. It is an important putative target for the development of a novel strategy for clinical treatment and drug discovery for AD.

## Graphical Abstract

PTAFR exacerbates the neuronal inflammatory microenvironment in AD brain.

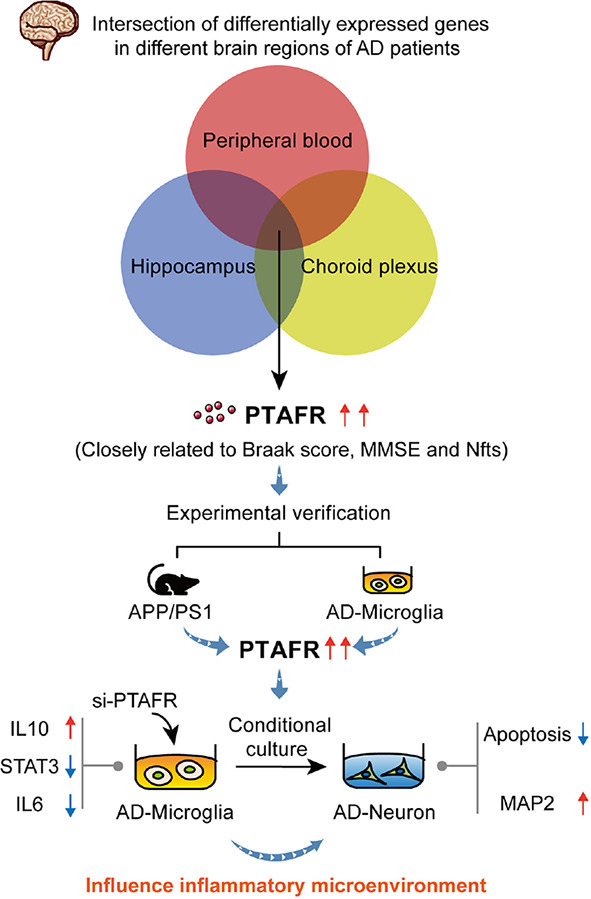

## Introduction

As a neurodegenerative disease closely associated with age, Alzheimer’s Disease (AD) seriously endangers the lives of the elderly (Robinson et al., 2017; Alzheimers Dementia, 2020). Cognitive deficits, memory loss, and language dysfunction are the major clinical characteristics of AD (McKhann et al., 2011). The hallmark pathophysiological changes include senile plaques formation by Amyloid β (Aβ) deposition in the brain, neurofibrillary tangles by tau hyperphosphorylation, and gliosis (Hyman et al., 2012; Kerbler et al., 2015). After the occurrence of the first clinical symptoms, the progression is harder to reverse (Sperling et al., 2011; Hane et al., 2017). Therefore, early diagnosis and treatment are the keys to delaying AD progression.

The clinical methods commonly used for AD diagnosis include Mini mental state examination (MMSE), 18 Fluorodeoxyglucose-positron emission tomography (^18^FDG-PET), Computed Tomography (CT), or Magnetic Resonance Imaging (MRI), Electroencephalogram (EEG), and evaluation of biomarkers in cerebrospinal fluid (CSF) of AD patients. Although the MMSE score is a convenient and low-cost method, it is often used for the diagnosis of moderate-to-severe AD. ^18^FDG-PET, CT, or MRI scanning are often used for excluding the possibilities of other diseases by imaging, thereby improving the reliability of AD diagnosis; however, these are inconvenient and expensive methods. EEG is insensitive in detecting early AD. The changes in biomarker expressions in the cerebrospinal fluid of AD patients seem to be more reliable for AD diagnosis, however, the sample collection procedure is invasive as compared to drawing other body fluids such as blood or urine (Hane et al., 2017; Ma et al., 2019). Therefore, identifying potential biomarkers having high specificity in the blood would be effective for the early diagnosis of AD.

Ideal AD biomarkers should be able to predict the incipient pathophysiological changes in brains and CSF of AD patients; they should be simultaneously detectable in peripheral body fluids, such as blood. High sensitivity and convenience for detection are necessary. As the brain tissues of AD patients are hard to obtain, this information is very limited. Therefore, in this study, we used GEO datasets to analyze the differentially expressed genes (DEGs) in AD brains, CSF, and blood. Finally, we identified a significantly upregulated DEG, Platelet activating factor receptor (PTAFR), which was closely related to AD progression. Furthermore, its predictive efficacy and the possible mechanism underlying AD were investigated and validated in the APP/PS1 mouse model and LPS+Aβ-induced BV2 cells.

## Materials and Methods

### Gene Expression Omnibus Data Collection

We searched for ‘‘AD’’ in GEO database,^1^ and microarray datasets GSE1297 from the GPL96 platform, GSE63063 chip from the GPL10558 platform and the GSE110226 chip from the GPL10379 platform were downloaded. In GSE1297, according to the MMSE score, hippocampal samples were divided into control group (MMSE > 26, *n* = 9), incipient AD group (MMSE: 20–26, *n* = 7), moderated AD group (MMSE: 14–19, *n* = 8), and severe AD group (MMSE < 14, *n* = 7). GSE63063 contained 135 control samples and 139 AD patients’ blood samples, while GSE110226 included six samples of normal choroid plexus and seven samples of choroid plexus from AD patients. See the Supplementary Material form for detailed patient information.

### Conversion and Difference Analysis of Raw Data

The GEO2R interactive online tool^2^ was applied to convert the raw data into a recognizable format. The differential expression genes (DEGs) were identified with *P* < 0.05 and | logFC| >1 as threshold values. The expression of genes obtained by VENN intersection was corrected by Bonferroni method.

### GO and KEGG Pathway Enrichment Analysis and Protein Interaction Analysis

The online enrichment platform David^3^ was used to conduct enrichment analysis of kyoto encyclopedia of genes and genomes (KEGG) pathway and gene ontology (GO) function on DEGs and screen the top-10 significant biological pathways with *P* < 0.05. Then, we used the bisoGenet plug-in in Cytoscape3.6.1 software to analyze the protein interaction of DEGs. Simultaneously, the protein interactions with the target gene *PTAFR* were evaluated through the String online tool.^4^

### Brain Samples and Blood Samples Collection

C57BL/6 mice were obtained from the Experimental Animal Center of China Medical University (Shenyang, China), and APP/PS1 transgenic mice were obtained from Jackson laboratory (Maine, USA). All animal care and experimental procedures were in compliance with the “Ethical Standards for Animal Laboratory Animals” of China Medical University. We collected the hippocampus of 12-month-old APP/PS1 mice (*n* = 5) and 12-month-old C57BL/6 mice (*n* = 5). We chose to study only female mice because incidence of AD is biased toward female (Jiao et al., 2016). The right hemisphere was used for Western Blotting, while the left hemisphere was immersed in paraformaldehyde for immunofluorescence staining. Blood was obtained from the orbit. Then, the blood samples were centrifuged at 3,000 rpm for 10 min at room temperature. The bottom layer was collected and used for RNA extraction.

### Cell Culture

The cells used in our experiment are BV2, SH-SY5Y, and SVGp12. Mouse-derived microglial BV2 cells and human-derived neuroblastoma SH-SY5Y cells were purchased from the Institute of Basic Medicine Chinese Academy of Medical Sciences (Beijing, China) while human-derived astrocytes SVGp12 were purchased from Bei Na Chuang Lian company (Beijing, China). Both BV2 and SVGp12 cells were cultured in DMEM medium (Invitrogen, Chicago, IL, United States), and SH-SY5Y cells were cultured in DMEM/F12 medium (Invitrogen, Chicago, IL, United States). All cells were supplemented with 10% fetal bovine serum (Thermo Fisher Scientific, Waltham, MA, United States) and 100 U/ml penicillin-streptomycin (Solarbio, Beijing, China), and placed them in a 37^°^C, 5% CO_2_ incubator for culture. The specific cell experiments are as follows. Both SVGp12 and SH-SY5Y were treated with 20 μM Aβ for 48 h to establish AD models. BV2 cells were treated with 1 μg/ml of LPS (Sigma-Aldrich, St. Louis, MO, United States) and 10 μM of Aβ (Sigma-Aldrich, St. Louis, MO, United States) for 48 h to establish AD inflammation model. The supernatant of BV2 treated in each group was added to Aβ-treated SH-SY5Y cells for 48 h for SH-SY5Y conditioned culture experiments. Cells of passage 5–20 were used for experiments.

### Transfection and Treatment of BV2 Cells

We transfected the si-*PTAFR* plasmid (Sangon Biotech, Shanghai, China) into BV2 cells as required, and the transfection reagent was Lipofectamine 3000 (Thermo Fisher Scientific, Waltham, MA, United States). In the nucleotide sequence of si-*PTAFR*, the sense strand is GCUAUGGGUCUUUGCUAACUUTT; the anti-sense strand is AAGUUAGCAAAGACCCAUAGCTT. When the cell density was about 60–70%, cells were transfected by using Lipofectamine 3000 according to the manufacturer’s instructions for 24 h. 1 μg/ml of LPS and 10 μM of Aβ (Aβ25-35 were placed in a 37^°^C incubator for 7 days before use) were incubated for another 24 h, then we collected the corresponding proteins and mRNAs and stored them at –80^°^C. The specific operation flow chart is shown in Supplementary Figure 1.

### Real-Time Polymerase Chain Reaction

We used the reverse transcription kit (Eric Biotechnology Company, Shanghai, China) to reverse transcribe the extracted total RNA into complementary cDNA, and then conducted the qPCR kit (Eric Biotechnology Company, Shanghai, China) according to the instructions required by the system (5 μl SYBR, 0.2 μl upstream and downstream primers, 0.2 μl ROX, 1 μl cDNA, 3.4 μl DEPC water) for real-time quantitative polymerase chain reaction (PCR) detection. The primers listed in this article are PTAFR, IL10, STAT3, and IL6. All primers were obtained from Sangon Biotech (Shanghai, China). The primer sequences are shown in Table 1. The results were processed by 2^–ΔΔCT^ method to compare the relative expression of RNA.

**TABLE 1:** Primer sequence list.

Primer	Sequence (5’–3’)
PTAFR-Forward	GAGTTTCGATACACGCTCTTTC
PTAFR-Reverse	CAAGTTAGCAAAGACCCATAGC
IL10-Forward	TTCTTTCAAACAAAGGACCAGC
IL10-Reverse	GCAACCCAAGTAACCCTTAAAG
STAT3-Forward	TGTCAGATCACATGGGCTAAAT
STAT3-Reverse	GGTCGATGATATTGTCTAGCCA
IL6-Forward	CTCCCAACAGACCTGTCTATAC
IL6-Reverse	CCATTGCACAACTCTTTTCTCA
GAPDH-Forward	AGCCTCGTCCCGTAGACAAAA
GAPDH-Reverse	TGGCAACAATCTCCACTTTGC

### Western Blotting

The BV2 cells and brain tissue were homogenized with protein lysate containing protease inhibitors (Beyotime Biotechnology, Shanghai, China) and the proteins were extracted, then quantified with a BCA kit (Beyotime Biotechnology, Shanghai, China). Equal amounts of protein were separated by SDS-polyacrylamide gels and transferred to polyvinylidene fluoride membranes (Millipore, Bedford, MA, United States). Then, the membrane was incubated in a blocking solution (a mixture of TBST containing 0.1% Tween-20 and 5% BSA) for 1 h at room temperature and placed in a primary antibody containing PTAFR (Abcam104162, 1:200, Abcam, Cambridge, MA, United States), IL10 (Wanlei03088, 1:1,000, Wanlei, Shenyang, China), STAT3 (CST12640s, 1:1,000, CST, Boston, MA, United States), IL6 (Wanlei02841, 1:1,000, Wanlei, Shenyang, China), MAP2 (CST4542s, 1:1,000, CST, Boston, MA, United States), and Syn (CST4329s, 1:1,000, CST, Boston, MA, United States) at 4^°^C overnight. The next day, after washing with TBST, the membrane was incubated with the corresponding HRP secondary antibody (Proteintech, Chicago, IL, United States). The immune response band was observed by enhanced chemiluminescence (ECL) with luminescence and quantified by measuring the density of each band using Image-J software.

### CCK8 Detection

The cell survival viability was assessed by CCK8 assay (DOJINDO, Japan). First, SH-SY5Y cells were spread in a 96-well plate (*n* = 5,000 cells/well) and cultured for 24 h. Then, the medium was changed to the transfected and modeled BV2 cell supernatant for 1 h. One microgram/milliliter LPS and 10 μM Aβ were given, and the culture was continued for 24 h. After that, we changed the medium to serum-free DMEM medium containing 10% CCK8 reagent, 100 μl per well, and incubate at 37^°^C for 2 h. Finally, we used a microplate reader to detect the absorbance at 450 nm wavelength and calculated the cell viability.

### Flow Cytometry to Measure Apoptosis

For apoptosis assays, SH-SY5Y cells were seeded in a 6-well plate (*n* = 2 * 10^5^ cells/well) and cultured for 24 h. Then, the medium was changed to the transfected and modeled BV2 cell supernatant for 1 h. One microgram/milliliter LPS and 10 μM Aβ were given, and the culture was continued for 24 h. Cells were washed twice with pre-cooled PBS, incubated with Annexin V-FITC and PI (BD Biosciences, United States) in the dark. Ultimately, cell apoptosis was analyzed by flow cytometer.

### Immunofluorescence

BV2 cells and SH-SY5Y cells were inoculated on a sterile cover glass into 12-well plates. After transfection and conditioned culture, the cells were fixed with 4% paraformaldehyde at room temperature. After washing with PBS, the cells were permeabilized with 0.5% TritonX-100 (Sigma–Aldrich, United States) for 20 min. The cells and brain tissue were blocked with goat serum (Boster Biological Technology, Wuhan, China) and stained with PTAFR or MAP2 in a humid box at 4^°^C overnight. Afterward, they were incubated with TRITC-conjugated rabbit anti-goat IgG (Thermo Fisher Scientific, Waltham, MA, United States) for 1 h at 37^°^C in a dark and humid box, followed by counterstaining with DAPI (Beyotime Biotechnology, Shanghai, China). Finally, the immunofluorescence image was obtained by laser scanning of a confocal microscope.

### Molecular Operating Environment Molecular Docking

The protein secondary structure of PTAFR were downloaded from the PDB website^5^ while the three-dimensional structure of EGCG, donepezil, curcumin, memantine, and Huperzine A were acquired from the PubChem website.^6^ Then, we imported the protein and small molecule drug structure into the molecular operating environment (MOE) (2018 version) software and convert the protein secondary structure into the tertiary structure. Finally, we performed molecular docking between protein and drugs after the small molecule drug is optimized.

### Statistical Analysis

All data were statistically analyzed using GraphPad Prism 8.0.1 version. The data were expressed as mean ± standard deviation. Differences between groups were evaluated by one-way analysis of variance. All determinations were repeated three times. *P* < 0.05 was considered statistically significant.

## Results

### Thirty Two Differential Expression Genes Are Closely Related to Alzheimer’s Disease Progression in the GSE1297 Cohort

**FIGURE 1:**
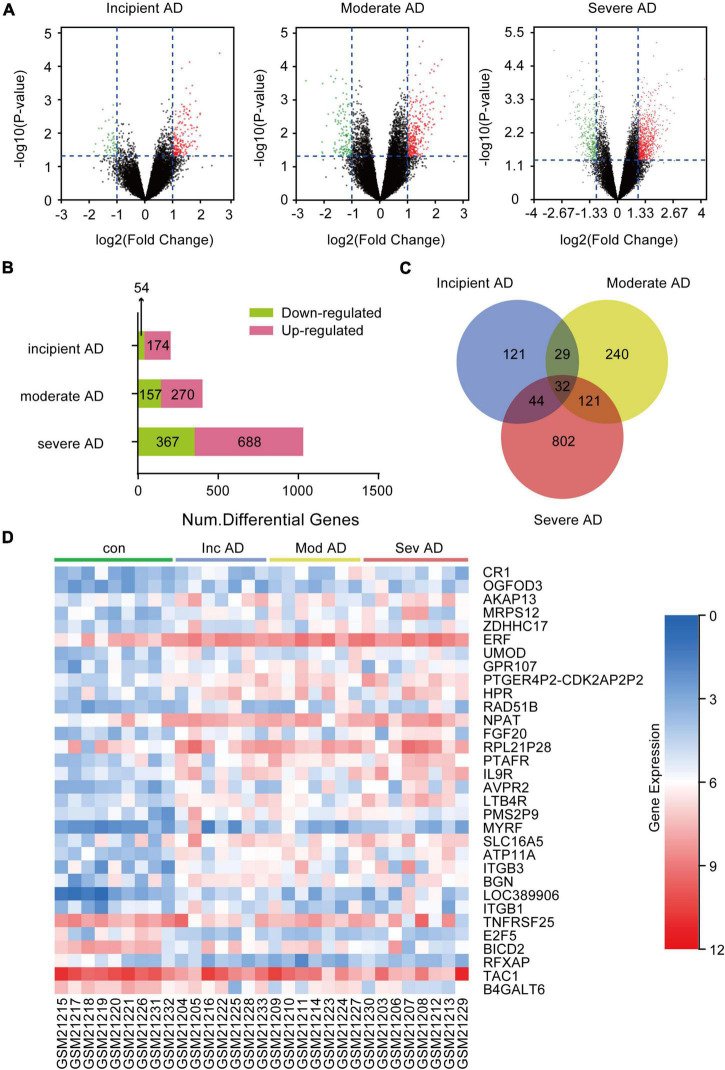
Differentially expressed genes (DEGs) in the hippocampus of Alzheimer’s Disease (AD) patients from GSE1297. GSE 1297 included the expression profiles of hippocampal CA1 tissues from 22 postmortem AD samples at different stages of severity. Seven, eight, and seven subjects were diagnosed with incipient, moderate, and severe AD, respectively. The DEGs were identified using the set criteria of |logFC| > 1 and *P* < 0.05. **(A)** Volcano maps for DEGs in the incipient, moderate, and severe AD samples. **(B)** The distribution of up-regulated and down-regulated DEGs in each stage. **(C)** Thirty two DEGs are common to the different AD stages as shown in the VENN map. **(D)** Heat map for distribution of the 32 DEGs.

To obtain the DEGs related to AD progression, we extracted the GSE 1297 chip from the GEO database. The GSE 1297 included the expression profiling of the hippocampus from 22 postmortem brain samples of AD patients at different stages of severity (incipient, moderate, and severe AD). The DEGs were identified with the set criteria of | logFC| >1 and *P* < 0.05, as shown in Figure 1A. A total of 174 DEGs were up-regulated, while 54 were down-regulated in incipient AD samples; 270 were up-regulated, while 157 were down-regulated in the moderate AD samples, and 688 were up-regulated, while 367 were down-regulated in severe AD samples (Figure 1B). Next, using the VENN graph network tool,^7^ the intersecting DEGs that were significant in all the stages of AD were obtained; a total of 32 DEGs were overlapping (Figure 1C and Table 2). They were further analyzed by plotting a heat map using the Sangerbox program (Figure 1D). A total of 25 genes were significantly up-regulated spanning across the incipient, moderate, and severe stages of AD, including, *CR1, OGFOD3, AKAP13, MRPS12, ZDHHC17, ERF, UMOD, GRP107, PTGER4P2-CDK2AP2P2, HRP, RAD51B, NPAT, FGF20, RPL21P28, PTAFR, IL9R, AVPR2, LTB4R, PMS2P9, MYRF, SLC16A5, ATP11A, ITGB3, BGN*, and *LOC389906*. A total of six genes, including *TNFRSF25, E2F5, BICD2, RFXAP, TAC1*, and *B4GALT6* were significantly down-regulated consistently in each stage of AD. Only *ITGB1* was up-regulated in the incipient and moderate AD stages, whereas down-regulated in the severe AD stage (Table 2). Therefore, based on the characteristics of biomarkers, we focused on the 25 up-regulated DEGs consistently in each stage of AD.

**TABLE 2:** Differential expression genes (DEGs) in the brain hippocampus of incipient, moderate, and severe Alzheimer’s Disease (AD) in GSE1297.

Gene name	Gene description	Incipient AD		Moderate AD		Severe AD	
		*P*-Val	logFC	*P*-Val	logFC	*P*-Val	logFC
CR1	Complement component 3b/4b receptor 1 (Knops blood group)	0.042	1.04	0.006	1.62	0.028	1.10
OGFOD3	2-oxoglutarate and iron dependent oxygenase domain containing 3	0.038	1.06	0.007	1.02	0.003	1.46
AKAP13	Kinase anchoring protein 13	0.031	1.11	0.035	1.08	0.001	1.29
MRPS12	Mitochondrial ribosomal protein S12	0.038	1.11	0.010	1.40	0.005	1.72
ZDHHC17	Zinc finger DHHC-type containing 17	0.017	1.15	0.001	1.44	0.007	1.03
ERF	ETS2 repressor factor	0.003	1.18	0.001	1.44	0.001	1.38
UMOD	Uromodulin	0.024	1.20	0.026	1.09	0.013	1.16
ITGB1	Integrin subunit beta 1	0.038	1.25	0.044	1.38	0.001	–1.39
GPR107	G protein-coupled receptor 107	0.021	1.26	0.018	1.46	0.029	1.21
PTGER4P2-CDK2AP2P2	PTGER4P2-CDK2AP2P2 readthrough, transcribed pseudogene	0.001	1.32	0.001	1.48	0.003	1.40
HPR	Haptoglobin-related protein	0.025	1.32	0.033	1.22	0.002	1.77
RAD51B	RAD51 paralog B	0.005	1.33	0.016	1.21	0.005	1.23
NPAT	Nuclear protein, coactivator of histone transcription	0.002	1.37	0.012	1.11	0.004	1.35
FGF20	Fibroblast growth factor 20	0.002	1.48	0.006	1.32	0.016	1.44
RPL21P28	Ribosomal protein L21 pseudogene 28	0.017	1.52	0.008	1.41	0.022	1.53
PTAFR	Platelet activating factor receptor	0.007	1.53	0.000	1.90	0.000	2.35
IL9R	Interleukin 9 receptor	0.003	1.53	0.009	1.09	0.000	1.98
AVPR2	Arginine vasopressin receptor 2	0.003	1.55	0.001	1.64	0.005	1.74
LTB4R	Leukotriene B4 receptor	0.000	1.58	0.003	1.29	0.001	1.76
PMS2P9	PMS1 homolog 2, mismatch repair system component pseudogene 9	0.006	1.64	0.002	1.60	0.023	1.16
MYRF	Myelin regulatory factor	0.018	1.66	0.002	1.43	0.001	1.53
SLC16A5	Solute carrier family 16member 5	0.010	1.67	0.014	1.47	0.008	1.65
ATP11A	ATPase phospholipid transporting 11A	0.000	1.68	0.000	1.85	0.002	1.50
ITGB3	Integrin subunit beta 3	0.006	1.86	0.010	1.86	0.018	2.02
BGN	Biglycan	0.002	1.96	0.016	1.47	0.020	1.59
LOC389906	Zinc finger protein 839 pseudogene	0.000	2.66	0.005	1.70	0.000	2.64
TNFRSF25	TNF receptor superfamily member 25	0.027	–1.11	0.019	–1.13	0.044	–1.30
E2F5	E2F transcription factor 5	0.023	–1.35	0.005	–1.53	0.027	–1.29
BICD2	BICD cargo adaptor 2	0.020	–1.16	0.011	–1.21	0.004	–1.86
RFXAP	Regulatory factor X associated protein	0.018	–1.13	0.004	–1.49	0.012	–1.15
TAC1	Tachykinin precursor 1	0.021	–1.08	0.007	–1.39	0.011	–1.50
B4GALT6	Beta-1,4-galactosyltransferase 6	0.032	–1.06	0.002	–1.46	0.002	–1.41

**FIGURE 2:**
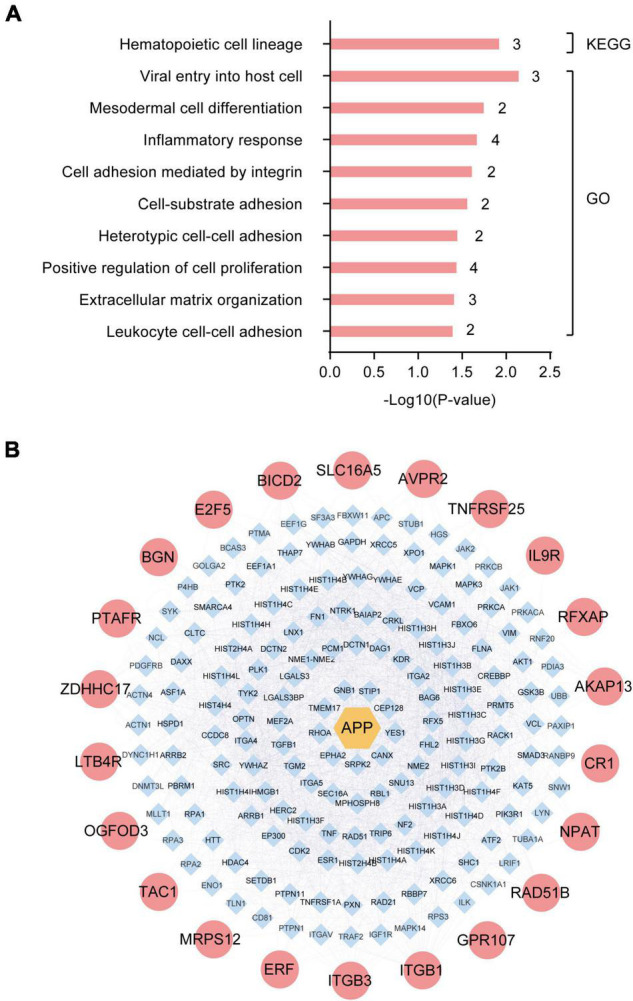
Enrichment and protein-protein interaction (PPI) network analysis of differentially expressed genes (DEGs) in the hippocampus of Alzheimer’s Disease (AD) patients from GSE1297. **(A)** The top-10 statistically significant biological processes related to the 32 DEGs based on KEGG pathway enrichment and GO functional annotation analyses. **(B)** Protein-protein interactions between the pathogenic gene, APP, and the 32 DEGs using the bisoGenet plug-in of the Cytoscape3.6.1 software.

To identify the dominant pathways related to the DEGs and their biological roles, we performed enrichment analysis using the KEGG pathway enrichment and GO annotation using the David network enrichment tool (see text footnote 3). The top-10 statistically significant biological pathways were obtained (Figure 2A). The biological processes, included hematopoietic cell lineage, viral entry into host cells, mesodermal cell differentiation, inflammatory response, cell adhesion mediated by integrin, cell-substrate adhesion, heterotypic cell-cell adhesion, positive regulation of cell proliferation, extracellular matrix organization, and leukocyte cell-cell adhesion. Next, we also evaluated the interactions between the 32 DEGs using the bisoGenet plug-in in the Cytoscape 3.6.1 software. A total of 22 genes showed possible interactions with the core pathogenic gene, APP, including *PTAFR, BGN, E2F5, BICD2, SLC16A5, AVPR2, TNFRSF25, IL9R, RFXAP, AKAP13, CR1, NPAT, RAD51B, GPR107, ITGB1, ITGB3, ERF, MRPS12, TAC1, OGFOD3, LTB4R*, and *ZDHHC17* (Figure 2B).

### Platelet Activating Factor Receptor Is Upregulated in Peripheral Blood, Cerebrospinal Fluid, and Hippocampus of Alzheimer’s Disease Patients

**FIGURE 3:**
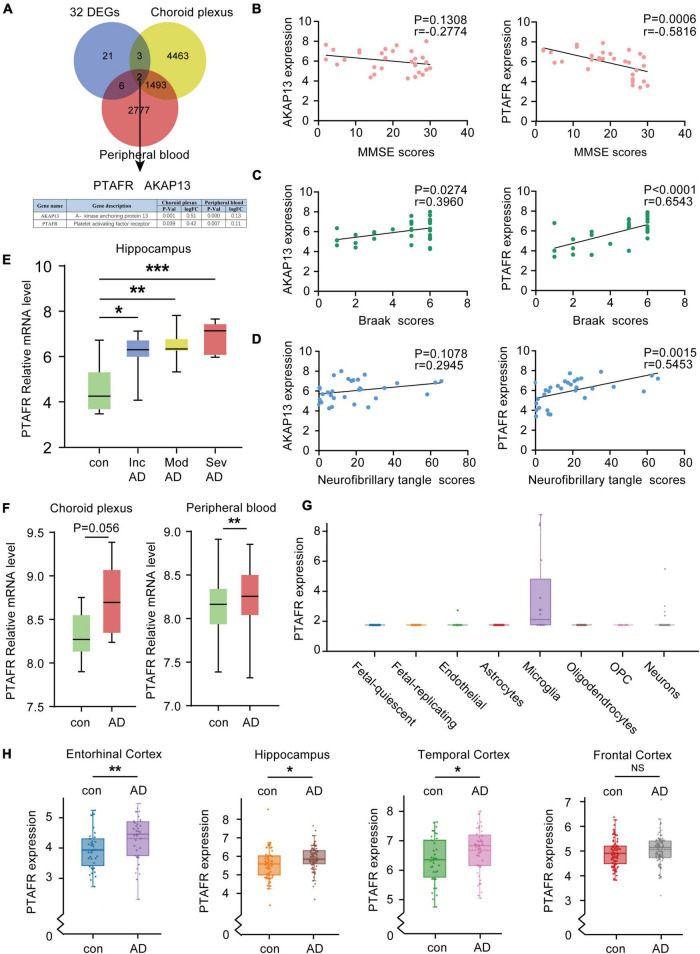
Platelet activating factor receptor (PTAFR) is specifically and highly expressed in the peripheral blood, cerebrospinal fluid, and hippocampus of Alzheimer’s Disease (AD) patients. **(A)** Analysis of overlapping differentially expressed genes (DEGs) in the hippocampus, cerebrospinal fluid (CSF), and peripheral blood from AD patients using a VENN map. **(B)** The correlations between the mRNA expressions of AKAP13 (left) or PTAFR (right) and MMSE scores are based on linear regression analysis. **(C)** The correlations between the mRNA expressions of AKAP13 (left) or PTAFR (right) and Braak scores are based on linear regression analysis. **(D)** The correlations between the mRNA expressions of AKAP13 (left) or PTAFR (right) and neurofibrillary tangle scores are based on linear regression analysis. **(E)** The mRNA expression of PTAFR in the AD hippocampus spans the AD progression stages. **(F)** PTAFR mRNA expression in cerebrospinal fluid and peripheral blood of AD samples. **(G)** PTAFR expression in different neuronal cells based on the AlzData platform. **(H)** The mRNA expression levels of PTAFR in the entorhinal cortex, hippocampus, temporal cortex, and frontal cortex. Error bars represent ± SD. *P < 0.05, **P < 0.01, ***P < 0.001, as compared with the control group.

Given the 32 DEGs in the hippocampus of AD patients in different stages, these were reasonably considered as potential biomarkers for AD diagnosis. The expressions of potential biomarkers significantly change with the occurrence and progression of AD, thus, the changes in DEGs in the hippocampus needed to synchronize with the changes in blood and cerebrospinal fluid (CSF). We used the CSF chip (GSE110226) and the blood chip (GSE63063) for further investigation. The VENN graph network tool was used to identify potential target DEGs simultaneously upregulated in blood, CSF, and hippocampus of AD patients. Finally, two DEGs, *PTAFR* and *AKAP13*, were obtained (Figure 3A). Next, the possible correlations between the mRNA expressions of *PTAFR* and *AKAP13* with MMSE scores were examined. We found that the mRNA expression of *PTAFR* was significantly correlated with the MMSE score (*P* = 0.0006), while that of AKAP13 was not statistically significant (*P* = 0.1308), shown in Figure 3B. Additionally, the Braak staging was more strongly correlated with *PTAFR* than AKAP13 (*PTAFR*, *P <* 0.0001; AKAP13, *P* = 0.0274; Figure 3C). We also found that the expression of *PTAFR* positively correlated with the neurofibrillary tangle scores, unlike AKAP13 (*PTAFR*, *P* = 0.0015; AKAP13, *P* = 0.1078; Figure 3D). The mRNA expression of *PTAFR* gradually increased with AD progression from incipient to severe (*P <* 0.05, Figure 3E), while AKAP13 did not exhibit this trend (Supplementary Figure 2A). Moreover, both PTAFR and AKAP3 were upregulated in the choroid plexus and peripheral blood (Figure 3F and Supplementary Figure 2A). *PTAFR* was also highly expressed in the entorhinal cortex, hippocampus, and temporal cortex of AD brains, while *AKAP13* showed high expression in the hippocampus only (Figure 3H and Supplementary Figure 2C). Furthermore, we investigated the possible mechanism underlying PTAFR involvement in AD progression. Using the AlzData online network platform^8^ (Xu et al., 2018), we found that only PTAFR was highly expressed in microglia, which contributes to the inflammatory responses in the pathogenesis of AD, while AKAP13 was expressed in many cell lines (Figure 3G and Supplementary Figure 2B). Taken together, our findings suggested that *PTAFR* may be a more important target biomarker having higher efficacy for AD diagnosis.

### Functional Validation of High Platelet Activating Factor Receptor Expression in 12-Month-Old APP/PS1 Mice and BV2 Cells

**FIGURE 4:**
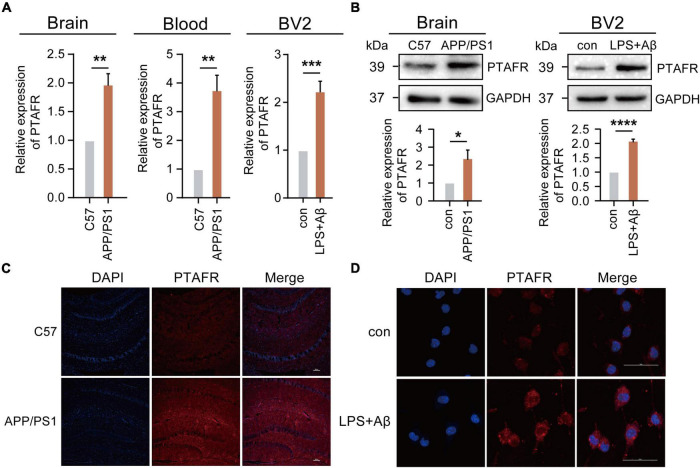
Platelet activating factor receptor (PTAFR) is highly expressed in 12-month-old APP/PS1 mice and LPS+Aβ-induced BV2 cells. **(A)** PTAFR mRNA expressions in the hippocampus and peripheral blood of C57BL/6 and APP/PS1 transgenic mice, and LPS+Aβ-induced BV2 cells. **(B)** Protein level expression of PTAFR in the hippocampus of C57BL/6, APP/PS1 mice, and LPS+Aβ-induced BV2 cells. **(C)** PTAFR protein expression in the hippocampus of C57BL/6 and APP/PS1 mice by immunofluorescence staining. **(D)** PTAFR expression in LPS+Aβ-induced BV2 cells. Error bars represent ± SD. *P < 0.05, **P<0.01, ***P < 0.001, ****P < 0.001, as compared to the C57BL/6 group or control group.

Our previous results using the GEO database indicated that PTAFR was the most relevant potential biomarker, which was highly expressed in AD patients. Thus, we validated its expression both *in vivo* and *in vitro*. We performed the assays using a 12-month-old APP/PS1 double transgenic AD mouse model. Indeed, PTAFR expression in APP/PS1 mice was significantly upregulated both at the mRNA and protein levels (*P* < 0.01, Figures 4A–C), as compared to the age-matched C57 BL/6J control mice. The peripheral blood levels of PTAFR in APP/PS1 mice also showed a significant increase (*P* < 0.01, Figure 4A).

Next, we validated these results *in vitro*. As mentioned previously, the predictive results based on the AlzData platform showed that PTAFR was specifically and highly expressed in microglia as compared to other neural cells in the central nervous system. A homologous sequence alignment of murine and human PTAFR genes showed high homology between the two (Supplementary Figure 3C). Therefore, we examined the PTAFR mRNA and protein levels in different cells, including the BV2 (microglia-like), SH-SY5Y (neuron-like), and SVGp12 (astrocyte-like) cell lines. We found that LPS+Aβ induction significantly enhanced the mRNA and protein level expressions of PTAFR in the BV2 cells (*P* < 0.01, Figures 4A,B,D), while there were no significant changes in the SVGp12 or SH-SY5Y cells as compared to the corresponding control groups (*P >* 0.05, Supplementary Figures 3A,B), which indicated that PTAFR was highly expressed in the microglia.

**FIGURE 5:**
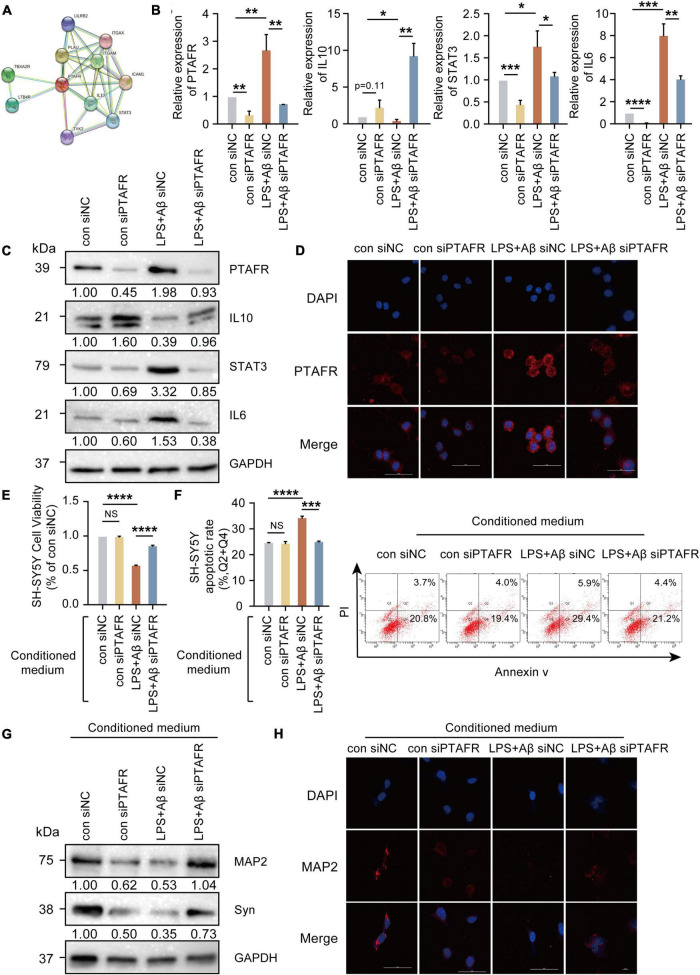
Platelet activating factor receptor (PTAFR) exaggerates the microglia-mediated microenvironment by increasing the inflammatory factors through IL10-STAT3 signaling. **(A)** PPI network diagram to predict the putative proteins interacting with PTAFR using the STRING online tool. **(B)** The mRNA expressions of PTAFR, IL10, STAT3, and IL6 in BV2 cells after silencing. **(C)** The protein expression levels of PTAFR, IL10, STAT3, and IL6 in BV2 cells after PTAFR silencing. **(D)** Immunofluorescence staining for PTAFR (red) and DAPI (blue) in BV2 cells. The conditional medium of BV2 cell was used to culture SH-SY5Y cells treated with LPS+Aβ. **(E)** Cell viability of SH-SY5Y cells after conditional medium (CM) treatment by CCK8 assay **(F)** Neuronal apoptosis in SH-SY5Y cells after CM treatment as detected by flow cytometry. **(G)** The expressions of MAP2 and Syn by western blotting. **(H)** Immunofluorescence staining of MAP2 (red) and DAPI (blue) in SH-SH5Y cells treated with CM. Error bars represent ± SD. *P < 0.05, **P < 0.01, ***P < 0.001, ****P < 0.001, as compared to the control group.

*PTAFR* is closely associated with the secretion of inflammatory factors upon kidney injury and in retinal neovascularization (Latchoumycandane et al., 2015a,b; Bhosle et al., 2016), indicating that neuroinflammation mediated by microglia may be the possible mechanism underlying PTAFR involvement in AD progression. The specific experimental flow chart is shown in Supplementary Figure 4. First, we found that PTAFR was closely associated with the inflammatory factors IL-10 and STAT3 using the STRING online tool (see text footnote 4) (Figure 5A). Extensive studies show that the IL10-STAT3 pathway is involved in the occurrence of many diseases (Zhang et al., 2017; Campana et al., 2018; Shirakawa et al., 2018; Cevey et al., 2019; Degboe et al., 2019; Wang et al., 2019). Thus, we reasonably hypothesized that PTAFR kindled the microglia-mediated neuroinflammation through the IL10-STAT3 signaling pathway and exaggerated the microenvironment of neurons in the progression of AD. After LPS and Aβ treatments, the mRNA and protein levels of IL-10 reduced significantly, while those of STAT3 and IL-6 were elevated, following PTAFR up-regulation (*P* < 0.05, Figures 5B–D). Further, we silenced the PTAFR gene in BV2 cells and found that the mRNA and protein levels of IL-10 increased, while those of STAT3 and IL-6 decreased substantially (*P* < 0.05, Figures 5B–D and Supplementary Figures 5A–F).

Next, the conditional medium (CM) of the treated BV2 cells was used to culture SH-SY5Y cells treated with LPS+Aβ to investigate the subsequent inflammatory efficacy of microglia on neurons. The CM of LPS+Aβ-induced BV2 cells significantly reduced the cell viability of SH-SY5Y cells (*P* < 0.0001, Figures 5E,F), while the CM of LPS+Aβ-induced BV2 cells after PTAFR silencing, could significantly improve the cell viability in SH-SY5Y cells; these findings were consistent in both CCK8 and Annexin V/PI flow cytometry assays (*P* < 0.001, Figures 5E,F). Moreover, the CM also remarkedly enhanced the expression of neuroplasticity indices, MAP2 and Syn (Figures 5G,H). Taken together, PTAFR, a potential biomarker, exaggerated the microglia-mediated microenvironment by upregulating inflammatory factors through IL10-STAT3 signaling.

### Targeted Docking of Platelet Activating Factor Receptor With Commonly Used Anti-Alzheimer’s Disease Drugs in Clinical Practice

**FIGURE 6:**
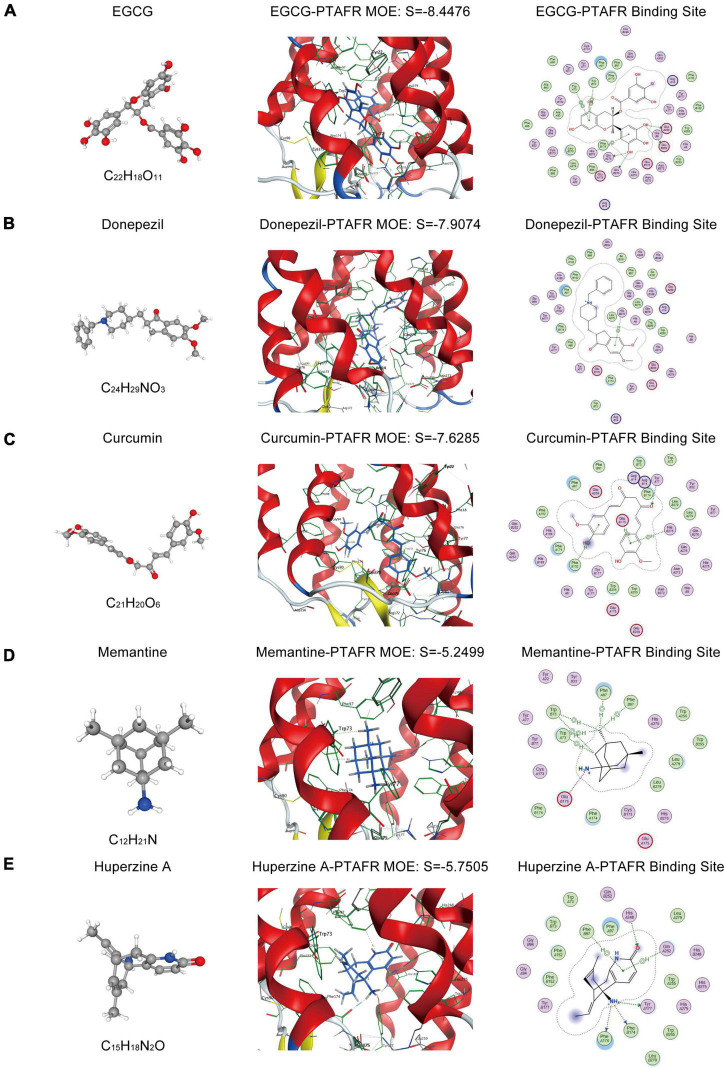
Targeted docking of platelet activating factor receptor (PTAFR) with Alzheimer’s Disease (AD) drugs commonly used in clinical practice. Panels **(A–E)** are the 3D structures of epigallocatechin gallate (EGCG), donepezil, curcumin, memantine, and Huperzine A, and their respective binding degrees and binding sites with PTAFR.

As described previously, PTAFR played an important role in exaggerating the microglia-mediated neuronal microenvironment through the IL10-STAT3 signaling pathway, which was closely correlated with AD progression. Therefore, it could be a potential biomarker or an essential target for the R&D of new anti-AD drugs. Thus, we performed targeted molecular docking of PTAFR with several drugs commonly used in clinical and scientific research for AD treatment, including donepezil, memantine, EGCG, curcumin, and Huperzine A. The molecular docking was performed using the MOE software. The S-value obtained after docking is used for evaluation of the possible binding; S < –7 was considered as having a significant probability of binding. EGCG is a potent compound that can exert anti-AD effects; the docking results showed that it could theoretically bind with PTAFR (S = –7.7826, Figure 6A). Donepezil is a commonly used medication used to treat mild-to-moderate AD. We also identified the possible binding sites (S = –7.5199, Figure 6B). Curcumin has neuroprotective efficacy and could bind to PTAFR, S = –7.5698 (Figure 6C). However, the S-values for memantine and Huperzine A were -5.3495 and S = –5.3781, respectively (Figures 6D,E). The compounds with planar structures and multiple benzene rings, such as EGCG, curcumin, and donepezil had a greater probability of binding to PTAFR, while those compounds showing stereo conformation, such as memantine and huperzine A, showed a significantly lesser probability of binding with PTAFR. These results indicated that PTAFR could bind to some anti-AD drugs and may have implications as a potential target for the treatment of AD in the future.

## Discussion

The pathophysiological changes in AD often precede the major clinical symptoms, such as cognitive dysfunction, as well as the characteristic pathological changes, including Aβ deposition and Tau hyperphosphorylation (Tan et al., 2014). Therefore, early diagnosis of AD and its intervention by targeting the initial stages will benefit the prognosis of patients and effectively improve their quality of life. At present, AD diagnosis mainly relies on MMSE score, ^18^FDG-PET, CT, or MRI scanning, and identification of T-tau, p-tau, Aβ42, and other biomarkers in the cerebrospinal fluid (Hane et al., 2017; Wolinsky et al., 2018; Guest et al., 2020). However, these methods have certain shortcomings, including high cost, lack of specificity, or invasive detection mode. Currently, the biomarkers of cerebrospinal fluid used in clinical settings include the detection of Aβ and Tau protein levels, however, this is often used for patients with advanced AD (McKhann et al., 2011). There remain certain doubts regarding the early diagnosis of AD. Nonetheless, AD, once developed, is difficult to reverse. When Aβ and Tau protein biological standards are used for detection, the best opportunity for early intervention is missed, thus, it is crucial to screen and identify early-stage molecular markers for AD and develop intervention strategies for early diagnosis and treatment of AD. In the present study, we identified a potential biomarker, PTAFR, by screening the GEO database and validating its efficacy both *in vivo* and *in vitro*.

An ideal AD biomarker should be able to detect early AD brain lesions based on peripheral body fluids, which correlated with brain lesions; it should be sensitive and easy to detect. Human brain tissue is extremely difficult to obtain, and ethical considerations further limit its use. Thus, we screened for potential candidates using the GEO database. A total of 32 DEGs related to the progression of AD were obtained based on the GSE1297 cohort; these were closely related to inflammation processes and 22 of them could interact with the core disease-causing gene, APP. Castillo et al. (2017) found that the expressions of genes that were closely related to inflammatory responses were significantly upregulated in APP^NL–GF/NL–GF^ mice. Venegas et al. (2017) shows that the activation of inflammasomes is closely related to the formation and progression of Aβ plaques in AD. Welikovitch et al.’s (2020) study shows that the neuron-specific inflammatory responses may occur earlier than the formation of Aβ plaques. Taken together, these studies suggest a close correlation between the inflammatory responses and Aβ deposition in end-stage AD, which was in line with our results. To identify ideal biomarkers reflecting the same changes in peripheral fluids as in the brain tissues, we further used the peripheral blood chip, GSE63063, and the choroid plexus chip, GSE110226. Finally, PTAFR was found to correlate with the severity of AD, indicated by its significant association with MMSE score, Braak staging, and neurofibrillary tangle scores, synchronously. However, no published report implicates the predictive utility of PTAFR for AD. Therefore, the PTAFR gene became the focus of our follow-up research. We performed functional verification using 12-month-old APP/PS1 mice and found that PTAFR was highly expressed in the brain and peripheral blood of APP/PS1 mice, consistent with our prediction results, which suggested that PTAFR was a potential candidate biomarker for AD diagnosis.

As a platelet-activating factor receptor, PTAFR plays an important role in several diseases. The expression of PTAFR in breast cancer cells and osteoclasts increases significantly, while, upon PTAFR downregulation, the breast cancer cell migration and osteoclast production reduce in the bone metastasis models (Hou et al., 2018). Moreover, PTAFR down-regulation is also correlated with the proliferation of cardiac fibroblasts and the deposition of collagen, which finally inhibits fibrous fibers after myocardial infarction in cardiac fibroblasts treated with angiotensin II (Zhao et al., 2020). However, PTAFR is rarely reported in the neurological field, and its role and the pathogenic mechanism underlying AD progression, remain unknown. Specifically, PTAFR was highly expressed in the microglia, while no significant increase in its expression was observed in astrocytes, oligodendrocytes, or neurons based on the AlzData website. Further, we also found PTAFR had specifically high expression in BV2 cells. Thus, we speculated that the function of the PTAFR gene in the central nervous system may be correlated with microglia-mediated biological processes. Microglia contributes to neuroinflammation and mediates the microenvironment of neurons. Previous studies show that PTAFR distributed on the surface of vascular endothelial cells can increase IL-1β expression, leading to inflammation-dependent vascular occlusion in the ischemic retinopathy model (Bhosle et al., 2016). In the kidney injury model, PTAFR is highly expressed and aggravates further upon kidney injury due to the induction of the expression of the inflammatory factor, TNFα (Latchoumycandane et al., 2015b). Additionally, in the renal fibrosis model induced by heavy ethanol intake, the expression of the inflammatory factor, TGFβ, and closely related indicators of renal fibrosis decrease significantly in the PTAFR knockout mice; finally, the renal fibrosis is inhibited (Latchoumycandane et al., 2015a). Furthermore, we also found that the expression of PTAFR was significantly increased in LPS+Aβ-induced BV2 cells, which suggested that PTAFR may be involved in the microglia-mediated inflammation of AD. However, almost no study reports the possible involvement of PTAFR in AD. We analyzed the putative protein interacting with PTAFR using the STRING online tool and found that IL10-STAT3 may be closely related to PTAFR. Our study showed an increase in the IL-10 expression, along with those of STAT3 and IL-6 upon PTAFR silencing in BV2 cells. After conditioned culturing of SH-SH5Y cells, their survival and plasticity were significantly enhanced after PTAFR silencing, which suggested that PTAFR may promote microglia-mediated neuroinflammation through the IL10-STAT3 signaling pathway and exaggerate the neuronal microenvironment in AD. In AD state, the inflammatory response of microglia has a certain positive regulatory effect on Aβ deposition, and we found that PTAFR was characteristically highly expressed in AD microglia and could affect the inflammatory responses of microglia. PTAFR may have a certain potential effect on the deposition of Aβ, in that, it can affect the inflammatory responses of microglia, thereby affecting the immune microenvironment of neurons and mediating the deposition of Aβ. IL-10 and STAT3 are involved in the occurrence and development of several diseases, such as AD, breast cancer, glioma, and autosomal dominant hereditary high lgE syndrome (Paulson et al., 2008; Yao et al., 2016; Chen et al., 2019; Porro et al., 2019). Kiyota et al.’s (2012) work shows that IL-10 significantly reduces neuroinflammation, enhances neuronal generation, and improves spatial cognitive impairment in APP/PS1 mice. Reichenbach’s work confirmed that APP/PS1 transgenic mice with STAT3 knockout have reduced Aβ plaque deposition in the brain, inhibited astrocyte proliferation, secretion of pro-inflammatory factors, and improved learning and memory (Reichenbach et al., 2019). The above studies suggest that IL-10 and STAT3 are involved in the AD inflammatory response, which supported our findings. Therefore, this study is the first report that investigates the relationship between PTAFR and IL10-STAT3 signaling in AD.

Since PTAFR may be a potential biomarker for AD diagnosis and a target closely related to inflammation, it could be used for the development of drug therapy and/or as a new anti-AD drug. For the first time, we performed MOE molecular docking between PTAFR and several drugs that are commonly used in clinical and scientific research for the treatment of AD and found that PTAFR could bind to some of these anti-AD drugs. Among them, PTAFR showed the highest binding degree with EGCG, donepezil, and curcumin, which indicated their likely interactions with PTAFR in therapy, thereby exerting anti-inflammatory effects in AD. The above results further highlighted the potential significance of PTAFR as an AD biomarker and a therapeutic target. Therefore, the further study of anti-AD drug treatment of APP/PS1 mice or LPS+Aβ-induced BV2 cells to reduce the expression of PTAFR and improve the inflammatory microenvironment around neurons has important research significance and value. These findings may provide a reference for the molecular design of new anti-AD drugs, however, further in-depth verifications are needed to confirm its clinical utility. In recent years, the identification of peripheral biomarkers closely related to neuroinflammation contributes to the early diagnosis and treatment of neurodegenerative diseases (Gambino et al., 2019). At present, there are mainly two kinds of peripheral inflammatory targets for AD disease, including Mannose-binding lectins (MBLs) and Fetuin-A protein. Studies have found that the expression level of MBLs protein in the cerebrospinal fluid of AD patients is significantly reduced (Moller-Kristensen et al., 2006), and the expression level of the anti-inflammatory factor Fetuin-A in the plasma of patients with mild to moderate AD is also significantly reduced (Smith et al., 2011). Inflammatory targets for Parkinson’s Disease (PD) disease currently include α-synuclein and vitamin D (Gambino et al., 2019; Li et al., 2021), both of which are also significantly down-regulated in the peripheral blood of PD patients. In addition, some studies have found that the blood brain barrier (BBB) of patients with neurodegenerative diseases is damaged, and the exosomes secreted by abnormally activated microglia can enter the peripheral blood through the BBB, and the contents contained in the exosomes can be detected (Guo et al., 2021). This helps in the early diagnosis of the disease.

In summary, PTAFR was identified as a potential biomarker for early AD diagnosis and treatment, which correlated with the microglia-mediated microenvironment. It may have implications as an important target for designing a novel strategy for clinical treatment and new drug discovery for AD. However, this study has certain limitations. We only found high expression of PTAFR in AD mouse brain and peripheral blood, but the specific function and role of PTAFR gene in AD still need to establish an AD mouse model lacking PTAFR gene for further in-depth research.

## Conclusion

In conclusion, a gene that was highly expressed in brain tissues, peripheral blood, and cerebrospinal fluid of AD patients, PTAFR was found to be a potential candidate biomarker for AD diagnosis. It was highly expressed in microglia and induced neuron inflammatory responses to exaggerate the microenvironment of AD neurons through IL10-STAT3 signaling *in vitro* experiments on cell lines. However, whether PTAFR can further interfere with the immune microenvironment around neurons by affecting the IL10-STAT3 pathway after silencing in the brain of APP/PS1 mice still needs further exploration. In addition, although we have used MOE software to dock PTAFR with various potential anti-AD drugs, whether the docked drugs can improve the inflammatory microenvironment in the brain of AD patients through the combination with PTAFR as we expected still need further in-depth study. Despite certain limitations, the findings may have implications for the development of a novel intervention target for AD treatment and a possible reference for the molecular design of new anti-AD drugs.

## Data Availability Statement

The original contributions presented in the study are included in the article/Supplementary Material, further inquiries can be directed to the corresponding authors.

## Ethics Statement

The animal study was reviewed and approved by China Medical University.

## Author Contributions

JL and ML designed and conceived the research, collected and analyzed the data, and wrote and revised the manuscript. JL and SL performed the cell experiments. JL, TM, and MX conducted the animal experiments. LJ, XZ, WY, KD, YWu, JT, WJ, YWa, MH, and WX directed the manuscript. All authors approved the final manuscript.

## Conflict of Interest

The authors declare that the research was conducted in the absence of any commercial or financial relationships that could be construed as a potential conflict of interest.

## Publisher’s Note

All claims expressed in this article are solely those of the authors and do not necessarily represent those of their affiliated organizations, or those of the publisher, the editors and the reviewers. Any product that may be evaluated in this article, or claim that may be made by its manufacturer, is not guaranteed or endorsed by the publisher.

## Footnotes

1
https://www.ncbi.nlm.nih.gov/geo/

2
https://www.ncbi.nlm.nih.gov/geo/geo2r

3
http://david-d.ncifcrf.gov/

4
https://string-db.org/

5
http://www1.rcsb.org/

6
https://pubchem.ncbi.nlm.nih.gov/

7
https://bioinfogp.cnb.csic.es/tools/venny/index.html

8
http://www.alzdata.org

## Abbreviations

18FDG-PET: 18 fluorodeoxyglucose-positron emission tomography
AD: Alzheimer’s Disease
APP/PS1: APPswe/PS1-de9
APP: amyloid precursor protein
Aβ: amyloid β
BBB: blood brain barrier
CSF: cerebrospinal fluid
CT: computed tomography
DEG: differential expression gene
EEG: electroencephalogram
EGCG: epigallocatechin gallate
GEO: gene expression omnibus
IL10: interleukin 10
IL6: interleukin 6
LogFC: log fold change
LPS: lipopolysaccharide
MAP2: microtubule-associated protein-2
MBLs: mannose-binding lectins
MMSE: mini mental state examination
MRI: magnetic resonance imaging
PD: Parkinson’s Disease
PTAFR: platelet activating factor receptor
siRNA: small interfer RNA
STAT3: signal transducer and activator of transcription 3
Syn: synaptophysin.
