# Supplementary material for: Platelet Activating Factor Receptor Exaggerates Microglia-Mediated Microenvironment by IL10-STAT3 Signaling: A Novel Potential Biomarker and Target for Diagnosis and Treatment of Alzheimer’s Disease
Source: Front Aging Neurosci. 2022 Apr 28;14:856628. doi: 10.3389/fnagi.2022.856628 (PMC9096237; doi:10.3389/fnagi.2022.856628)
Supplement: Supplementary file 1 [file Data_Sheet_1.docx]

***Supporting Information***


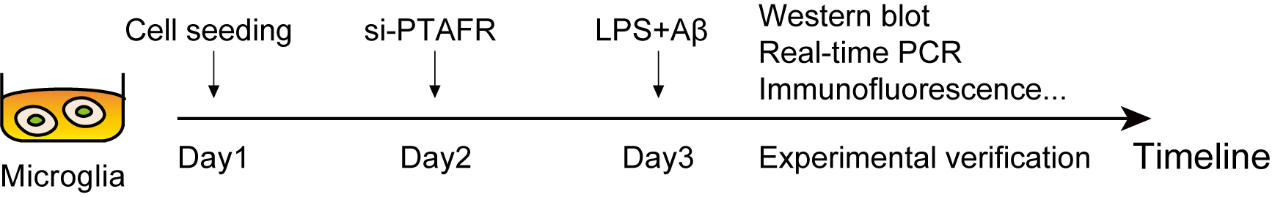


**FigS1. Transfection timeline of the PTAFR gene.**


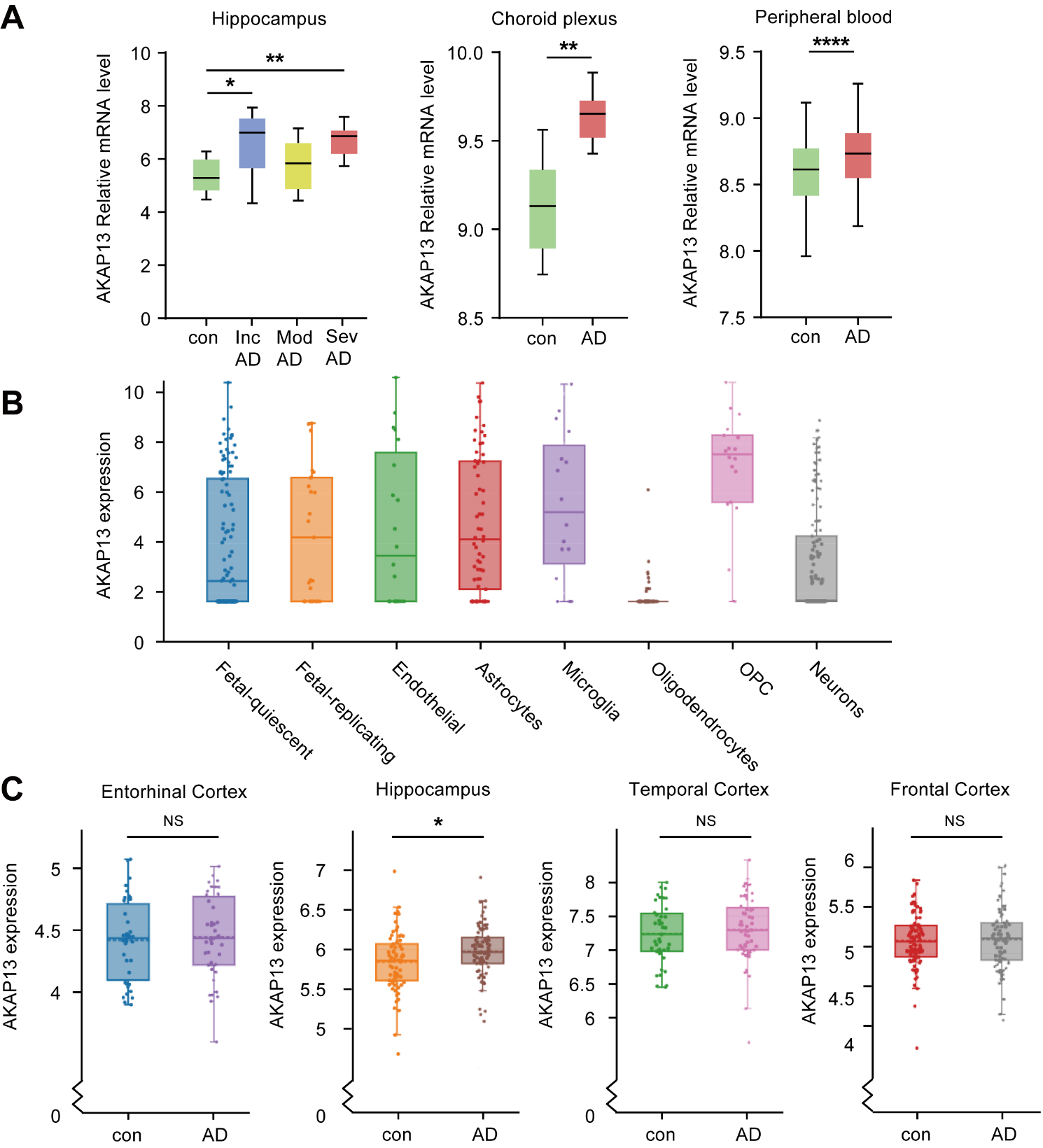


**FigS2. AKAP13 was highly expressed in peripheral blood, cerebrospinal fluid and hippocampus of AD patients.** (**A**) AKAP13 gene mRNA expression in hippocampus, cerebrospinal fluid and peripheral blood of AD patients. (**B**) AKAP13 expression in different types of neural cells in the AlzData platform. (**C**) The mRNA expression levels of AKAP13 in the entorhinal cortex, hippocampus, temporal cortex and frontal cortex, respectively. Error bars represent ± SD. ^*^*P*<0.05, ^**^*P*<0.01, ^***^*P*<0.001, ^****^*P*<0.001, compared with control group.


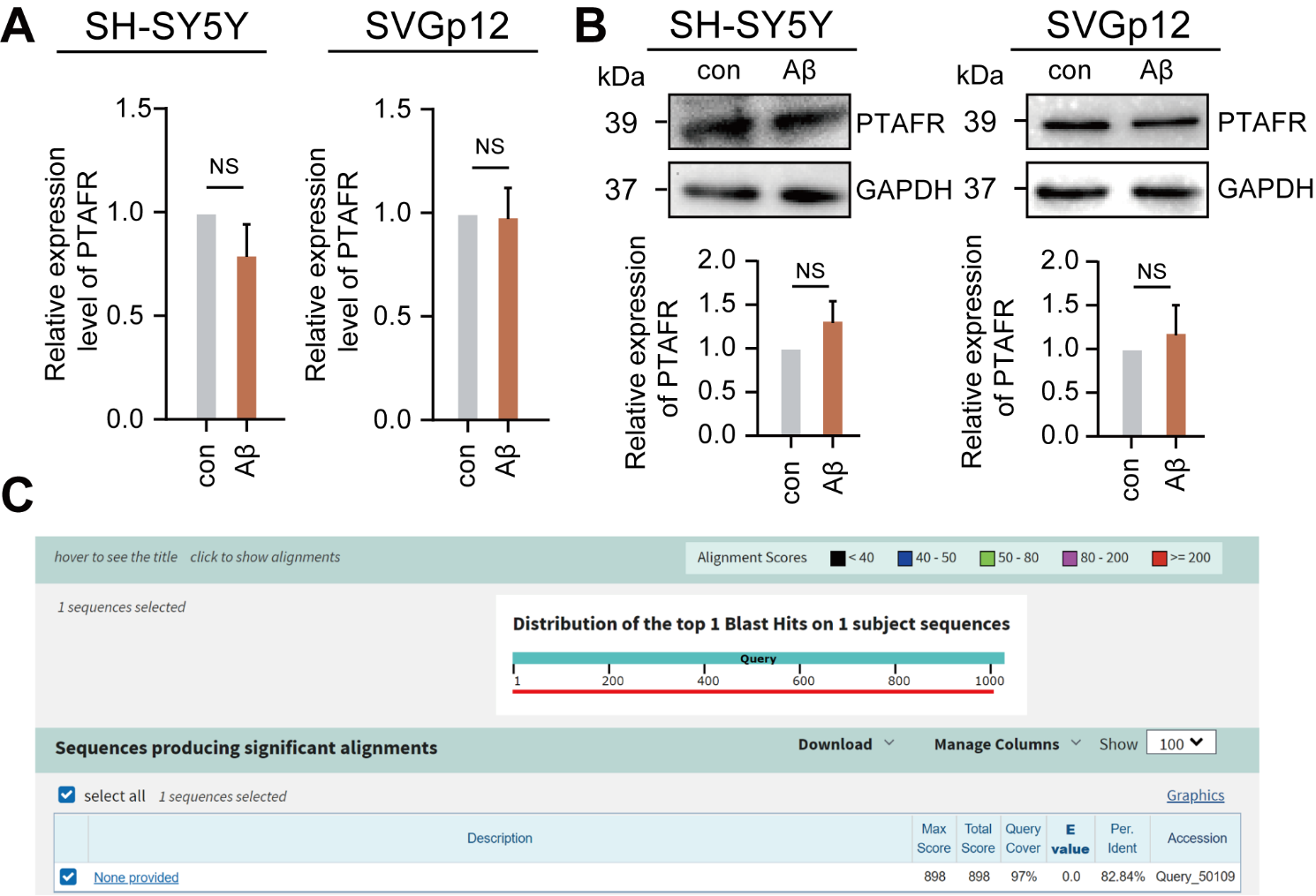


**FigS3. PTAFR expressions in SVGp12 and SH-SY5Y cells induced by Aβ.** mRNA expression (**A**) and protein expression (**B**) of PTAFR in SVGp12 and SH-SY5Y cells induced by Aβ, respectively. (**C**) Homologous sequence alignment of murine PTAFR gene and human PTAFR gene. Error bars represent ± SD. ^*^*P*<0.05, ^**^*P*<0.01, ^***^*P*<0.001, ^****^*P*<0.001, compared with control group.


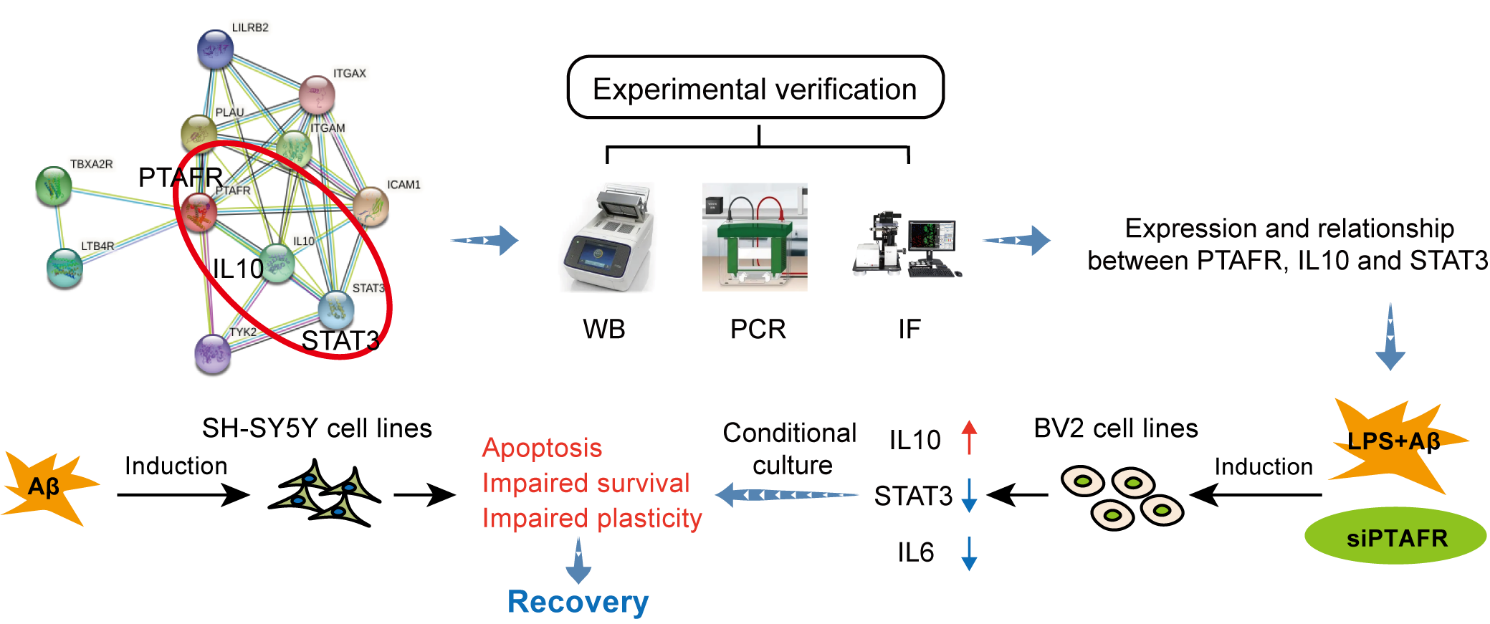


**FigS4. The schematic diagram of the experimental flow in Fig5.**


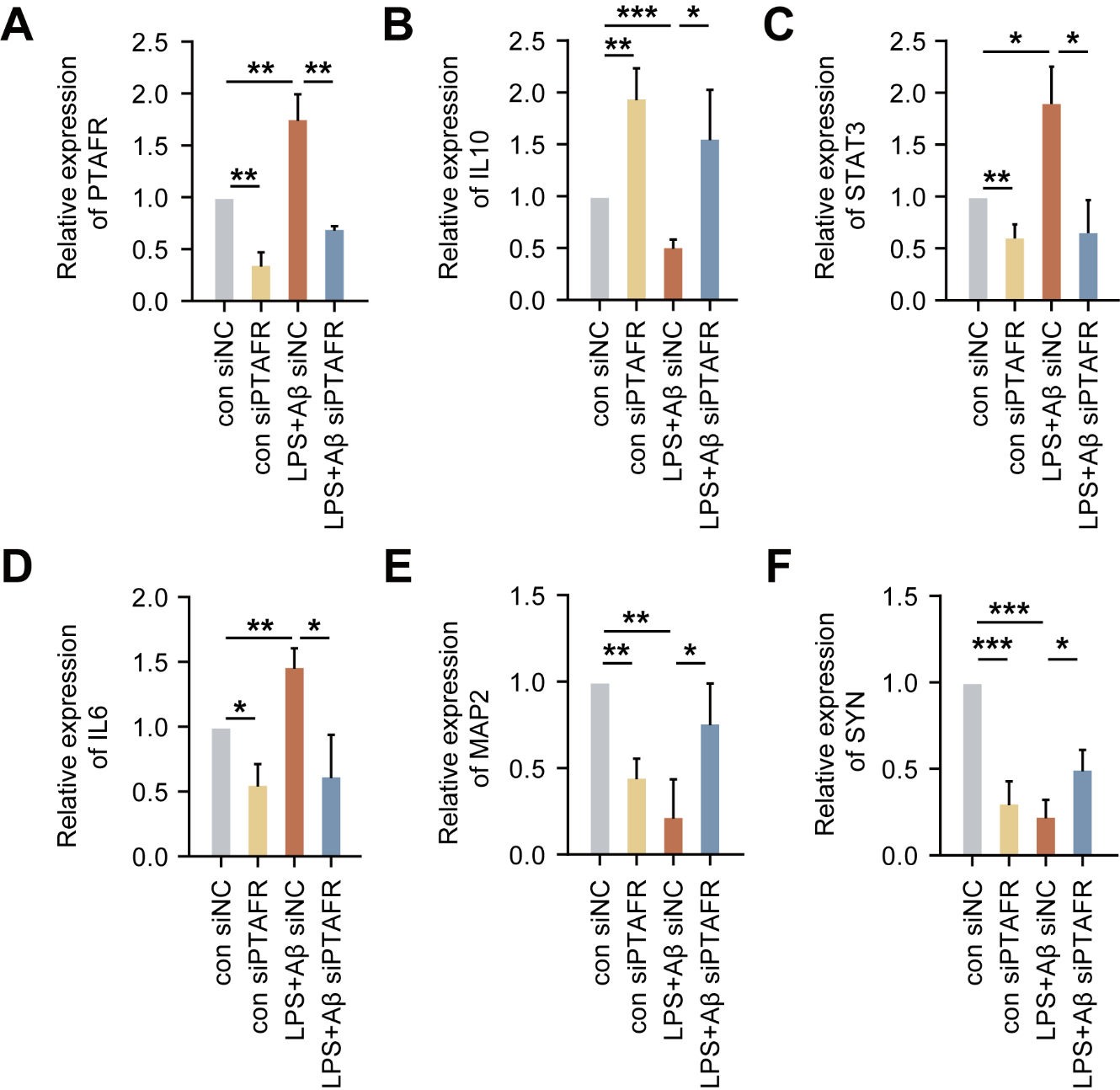


**FigS5. Statistical chart of Western Blot gray value of Fig5.** Protein expression of PTAFR (**A**), IL10 (**B**), STAT3 (**C**), IL6 (**D**) in BV2 and MAP2 (**E**), Syn (**F**) in SH-SY5Y. Error bars represent ± SD. ^*^*P*<0.05, ^**^*P*<0.01, ^***^*P*<0.001, compared with control group.
